# Qu-Zhuo-Tong-Bi Decoction Alleviates Gouty Arthritis by Regulating Butyrate-Producing Bacteria in Mice

**DOI:** 10.3389/fphar.2020.610556

**Published:** 2021-02-02

**Authors:** Xianghui Wen, Yu Lou, Siyue Song, Zhixing He, Juan Chen, Zhijun Xie, Xiaowei Shi, Chengping Wen, Tiejuan Shao

**Affiliations:** College of Basic Medical Science, Zhejiang Chinese Medical University, Hangzhou, China

**Keywords:** Qu-zhuo-tong-bi decoction, gouty arthritis, gut microbiota, SCFAs, gut homeostasis

## Abstract

Qu-zhuo-tong-bi decoction (QZTBD) is a traditional Chinese medicine prescription used to treat hyperuricemia and gout with no obvious adverse effects. However, the mechanism by which QZTBD treats gout has not been fully explored. Here, we investigated the effects of QZTBD on gouty arthritis and its therapeutic mechanism from the perspective of the gut microbiome. Our results demonstrated that QZTBD was effective for reducing serum uric acid level and attenuating paw edema and mechanical allodynia. QZTBD promoted the abundance of butyrate-producing bacteria and the production of SCFAs. Further study revealed that QZTBD restored the intestinal barrier function, modulated the expression of GPR43 and ABCG2, suppressed the activity of key glycolysis-related enzymes, and inhibited the generation of intestinal inflammatory factors. These findings suggested that QZTBD is an effective therapeutic drug for gouty arthritis. Butyrate-producing bacteria and its metabolites SCFAs might act as a potential target of QZTBD.

## Introduction

Gout is a purine metabolism disorder and chronic inflammatory disease with increasing prevalence and incidence, particularly in developed countries (Kuo et al., 2015). Severe pain, swelling, warmth, and redness at the gouty joint characterize the disorder (Dalbeth et al., 2016; So and Martinon, 2017; Yang et al., 2019). These traits develop following massive deposition of monosodium urate (MSU) crystals in joints and surrounding tissues. Establishing reliable treatments that target serum urate is essential for effective gout management. Allopurinol and febuxostat (FBST) are the first-line urate-lowering therapies (Dalbeth et al., 2016). However, common side effects of using these drugs in the clinical treatment of gout include skin rash, nausea, vomiting, diarrhea, abnormal liver function, and chronic renal toxicity. Strategies to achieve new treatment targets and to improve the quality of gout care are needed.

Traditional Chinese medicine (TCM) has been widely used in China for thousands of years. It is believed to be a valuable form of medicine with better efficacy and fewer side effects. Qu-zhuo-tong-bi decoction (QZTBD), an empirical prescription for gout treatment, has definite clinical effects on lowering serum urate levels and preventing the recurrence of gout attacks with no serious adverse effects. This prescription has obtained a national invention patent in China (Patent No.: ZL201010209598.7). Its multiple functions, including reinforcing renal function, promoting blood circulation, and relieving pain, have been demonstrated elsewhere (Chen et al., 2016; Lv et al., 2019). However, its underlying mechanism of its anti-gouty arthritis effect has not yet been fully explored.

A growing number of studies have shown that intestinal flora can participate in purine metabolism. Gut excretion of urate is critical for regulating serum urate (Ichida et al., 2012). Decreased gut urate excretion induced by a microbial imbalance is an important cause of gout (Guo et al., 2016). The gut microbiome has attracted more and more attention as a promising target for the treatment of gout. An increasing number of traditional Chinese herbal ingredients have been proven to manipulate the intestinal microbiota community structure. A fundamental way for TCM to take effect is the regulation of intestinal flora. To investigate the effect of QZTBD on intestinal flora and its potential mechanism in the treatment of gout, a high-fat diet (HFD) and MSU crystal-induced animal model was established. After treating the mouse model with QZTBD and FBST, the structure of intestinal flora was analyzed and the content changes of primary SCFAs in feces were detected. Our results showed that QZTBD can effectively attenuate gouty symptoms by restoring the gut microbiota and promoting the production of SCFAs, which is different from FBST.

## Materials and Methods

### QZTBD and Chemicals

QZTBD is composed of nine Chinese medicinal herbs (Table 1):*Smilax glabra* Roxb. (60 g, Batch No: 180701), *Dioscorea spongiosa* J. Q. Xi, M. Mizuno et W. L. Zhao (30 g, Batch No: 180701), *Zea mays* L. (15 g, Batch No: 180801), *Coix lacryma*-*jobi* L. var. *ma*-*yuen* (Rom.Caill.) Stapf (30 g, Batch No: 181001), *Siegesbeckia orientalis* L. (18 g, Batch No: 181001), *Taxillus chinensis* (DC.) Danser (15 g, Batch No: 180301), *Curcuma longa* L. (12 g, Batch No: 181101), *Corydalis yanhusuo* (Y. H. Chou and Chun C. Hsu) W. T. Wang ex Z. Y. Su and C. Y. Wu (18 g, Batch No: 180501), and *Citrus medica* L. var. *sarcodactylis* Swingle (12 g, Batch No: 180301). All herbal materials were purchased from Zhejiang Chinese Medical University Medical Pieces., LTD. (Hangzhou, China). The above plant samples were stored in a specific Herbarium room. QZTBD was prepared using a traditional decoction method. The mixed medicine was boiled with distilled water for 45 min, and the supernatant was collected by filtration. The filtrate was further concentrated using a rotary evaporator and dried using a freeze dryer. The final aqueous extracts of QZTBD were homogenized and stored in an air-tight container at −20°C. FBST and MSU were purchased from Hangzhou Zhuyangxin Pharmaceutical Co., Ltd (Hangzhou, China) and Sigma (MO, United States), respectively.

**TABLE 1:** The compositions of Qu-zhuo-tong-bi decoction (QZTBD).

Chinese name	Latin name	Scientific name	Weigh(g)	Parts used
Tu Fu Ling	*Rhizoma Smilacis Glabrae*	*Smilax glabra* Roxb	60	Rhizome
Bi Xie	*Dioscoreae Spongiosae Rhizoma*	*Dioscorea spongiosa* J. Q. Xi, M. Mizuno et W. L. Zhao	30	Rhizome
Yu Mi Xu	*Corn Stigma*	*Zea mays* L	15	Stigma
Yi Yi Ren	*Coicis Semen*	*Coix lacryma*-*jobi* L. var. *ma-yuen* (Rom.Caill.) Stapf	30	Seed
Xi Xian Cao	*Siegesbeckiae Herba*	*Siegesbeckia orientalis* L	18	Herb
Jiang Huang	*Curcumae Longae Rhizoma*	*Curcuma longa* L	12	Rhizome
Sang Ji Sheng	*Taxilli Herba*	*Taxillus chinensis* (DC.) Danser	15	Branch, Leaf
Yan Hu Suo	*Corydalis Rhizoma*	*Corydalis yanhusuo* (Y. H. Chou and Chun C. Hsu) W. T. Wang ex Z. Y. Su and C. Y. Wu	18	Tuber
Fo Shou	*Citri Sarcodactylis Fructus*	*Citrus medica* L. var. *sarcodactylis* Swingle	12	Fruit

### Quality Control and Content Determination of QZTBD

The main contents of QZTBD were analyzed by high-performance liquid chromatography (HPLC) for quality control of QZTBD. 0.2 g of dried QZTBD extract was resuspended in deionized water, sonicated for 30 min at 35 kHz and 25°C, and then centrifuged at 3,500 rpm for 5 min. The supernatant was filtered through a 0.22 μm PES filter before HPLC analysis. The HPLC analysis was carried out by Waters e2695 + 2489 HPLC system with C18-AR column (250 mm × 4.6 mm, 5 μm). The mobile phase consisted of acetonitrile (A) and 0.1% phosphoric acid (B). The elution program was as follows: 0–10 min 5–25% A; 10–20 min 25–30% A; 20–40 min 30–90% A; 40–42 min 90-5% A; 42–52 min 5-5% A. The flowrate was 1.0 ml/min, the detection wavelength was 220 nm, and the column temperature was maintained at 25°C.

Astilbin, tetrahydropalmatine, and quercetin are the main bio-active components of *Smilax glabra* Roxb., *Corydalis yanhusuo* (Y.H. Chou and Chun C. Hsu) W.T. Wang ex Z.Y. Su and C.Y. Wu, and *Taxillus chinensis* (DC.) Danser respectively. Rutin is the common component of Chinese medicinal herbs in the QZTBD. These four components were selected as the standards for quality control of QZTBD. All four chemicals (HPLC ≥ 98%) were purchased from Shanghai Yuanye Bio-technology Co., Ltd. (Shanghai, China) and were analyzed by the same elution program. The detection wavelength was at 220 nm, 360 nm, 360 nm, and 210 nm, respectively.

### Animals and Drug Administration

Twenty-eight specific pathogen-free male C57BL/6 mice (4–6 weeks, 15 ± 3 g) were obtained from Shanghai SLAC Laboratory Animal Co., Ltd. (Shanghai, China) and housed in the Laboratory Animal Center of Zhejiang Chinese Medical Animal Care (AAALAC) under standard environmental conditions (12 h light-dark cycles and 25 ± 1°C). Experimental procedures were approved by the Laboratory Animal Management and Welfare Ethical Review Committee of Zhejiang Chinese Medical University (Permission number: ZSLL-2018-0012).

After 1 week of acclimatization, the mice were randomly divided into four groups: control group, gouty arthritis model group, QZTBD group, and FBST group. Each group was comprised of seven animals. The control group was fed with a standard diet, and 40 μL of PBS was injected into the right hind footpad every 10 days. The animals in the other three groups were fed with HFD (10% yeast extract) and injected with MSU crystals (1 mg MSU crystals in 40 μL PBS/mouse) every 10 days to simulate gouty arthritis (Lin et al., 2020). Six-week therapy started at the induction of the gouty arthritis model. Drug dosages were calculated based on the conversions from clinical adult dosages and the body weight of mice. According to our previous study, the QZTBD group and the FBST group were administered by gavage with 18.0 g/kg/day QZTBD and 5.2 mg/kg/day FBST respectively (Liu et al., 2019). The control group and model group were given equal volumes of distilled water.

### Evaluation of Gouty Symptoms

At the end of the experiment, the blood was obtained by retro-orbital bleeding. The serum was then isolated. The concentration of serum uric acid (SUA) was detected strictly according to the operation steps of the TBA-40FR automatic biochemical analyzer (Toshiba Co., Ltd., Japan).

Footpad swelling was measured as reported previously (Lv et al., 2019). In brief, the footpad thickness for each mouse was measured by a digital caliper (Minet Industrial Co., Ltd., China) before stimulus and 4  h, 24  h, 48  h, and 72 h after the administration of MSU. Footpad swelling was evaluated as an increase in footpad thickness, which was calculated as the difference between the initial thickness and the test thickness observed at different time points. The pain threshold was measured by measuring the mechanical withdrawal threshold (MWT) with the *von* Frey monofilaments (Danmic Global Llc Co., Ltd., Unites States) (Yu et al., 2016; Lv et al., 2019). All the above behavioral tests were conducted by an investigator blinded to experimental conditions.

The claw region of mice was separated and fixed with 10% paraformaldehyde in PBS, decalcified for 3 weeks with EDTA, embedded in paraffin wax, and then stained with hematoxylin and eosin for conventional morphological evaluation.

### Analysis of Gut Microbiota

Feces were collected from the middle segment of the colon, and total DNA was extracted from stool samples using the QIAmp DNA microbiome kit (Qiagen, German) according to the manufacturer’s protocol. Gut microbiota abundance and diversity were analyzed by sequencing of 16S rRNA gene (V3-V4 region) with Illumina MiSeq platform. High-quality reads were selected and library size across samples were normalized to the same number to exclude the bias caused by different sequencing depth. The effective reads were aligned with the SILVA database and clustered into operational taxonomic units (OTUs) based on a 97% similarity cutoff using QIIME 2 (https://qiime2.org/). Alpha diversity and beta diversity analysis were performed by R software (1.2.5033). Bray-Curtis dissimilarity matrix was developed with the normalized sequences. Hierarchical cluster analysis (HCA), principal coordinates analysis (PCoA), and distance between two groups were performed based on the Bray-Curtis dissimilarity matrix. The linear discriminant analysis (LDA) effect size (LEfSe) method was applied to reveal the effect of each differentially abundant taxon and distinguish the key phenotypes responding to the QZTBD treatment, with a set logarithmic LDA score of 2.0. Spearman’s correlation coefficient was conducted between microflora and gout symptoms using the function “cor.test” in the statistical software R and visualized with a heatmap.

### Determination of Fecal Short-Chain Fatty Acids

The short-chain fatty acids (SCFAs) were extracted with anhydrous ether from acidified fecal water extract. The analysis was carried out using a gel filtration chromatography-mass spectrometry (GPC-GC/MS-2010, Shimadzu, Kyoto, Japan) with a Rtx-Wax capillary column (Shimadzu, Kyoto, Japan). A split injection of 1 µL sample was made at a ratio of 10:1, with a column helium flow rate of 1.2 ml/min. Ion source temperature was 230°C, while the injector temperature was 260°C. The column initial temperature was 100°C. It was then increased by 8°C/min to 140°C, which was held for 2 min. Column temperature was increased again by 60°C/min to 200°C and held there for 3 min. The scan mode was Scan and SIM mode. Identification of the primary SCFAs, including acetate, propionate, and butyrate, were carried out according to the retention time. SCFA standards acetate (#S5636), propionate (#P1880), and butyrate (#303410) were purchased from Sigma Aldrich. All samples were analyzed in triplicate.

### Immunohistochemical Analysis of Intestinal GPR43 and Tight-Junction Proteins

Formalin-fixed, paraffin-embedded colon tissue sections were prepared for immunohistochemistry (IHC) assays. Primary antibodies, including anti-ZO-1 (Affinity Biosciences, OH, United States, #AF5145), anti-Occludin (Affinity Biosciences, OH, United States, #DF7504), and anti-GPR43 (Bioss Inc., Mass, United States, #BS-13536R), were respectively applied to incubate with sections overnight at 4°C. After being incubated with anti-rabbit IgG for 60 min at room temperature, the stained sections were washed and visualized brown with DAB (C-0003, Bioss, Beijing, China) and scanned by a digital pathological section scanner (NDP, Hamamatsu, Japan). The staining was manually evaluated by two independent certified pathologists using NDP view 2.0 software.

### Real-Time *Quantitative* PCR (*q*PCR)

Total RNAs from colon tissue were extracted with Trizol reagent (Thermo Fisher Scientific, United States). The RNA was reverse transcribed into cDNA using random hexamers primers with HiFiScript cDNA Synthesis Kit (CWBIO, China) according to the manufacturer’s instructions. Each reaction was performed at least in triplicate and normalized to *β*-actin level. *q*PCR was performed with Bio-Rad iQ5 PCR system (Bio-Rad, United States) using the SYBR Premix ExTaq Kit (Takara Bio Inc., China). The CT value of each well was determined by instrument software. The average of the values was calculated. The relative quantification was analyzed by the 2^−ΔΔCt^ method. The primer sequences were shown in Supplementary Table S1.

### Western Blotting Analysis

The intestinal tissue was homogenized in RIPA protein lysis buffer (Thermo Fisher Scientific, United States) and the concentrations of the extracted proteins were measured using a BCA Protein Assay Kit (Beyotime Biotechnology, Shanghai, China). Protein was separated by 10% SDS-PAGE gel and transferred onto a PVDF membrane. The PVDF membrane was blocked with 5% milk in TBST buffer for 1 h at room temperature, probed with the primary antibodies overnight at 4°C, and then incubated with HRP-coupled secondary antibodies for 1 h at room temperature. The primary antibodies used in this study were anti-PFKFB3 (1:5000 dilution, Abcam, #ab181861) and anti-LDH (1:7500 dilution, Abcam, #ab52488). *β*-actin (1:1,000 dilution, Absin, #abs830031) was used to ascertain that equal amounts of protein were loaded. The gray value analysis of gel quantification was performed using ImageJ software.

### Lactic Acid Assay

The concentrations of serum and fecal lactic acid were determined using a lactic acid assay kit (Njjcbio, China; #A019-2-1) according to the manufacturer’s instructions. The absorbance of the samples was measured at 530 nm on a spectrophotometer. The concentrations of lactic acid were calculated according to the standard curve.

### Statistical Analysis

SPSS 20.0 was used to analyze the statistical difference of the experimental data. GraphPad Prism 7.0 software was used for drawing. The measurement data were expressed as mean ± SEM. The normality of the data and the homogeneity of variance between groups were tested using one-way ANOVA or the Wilcoxon rank-sum test. *p*-value below 0.05 was considered statistically significant.

## Results

### Quantitative Analysis of the Chemical Constituents of QZTBD

**FIGURE 1:**
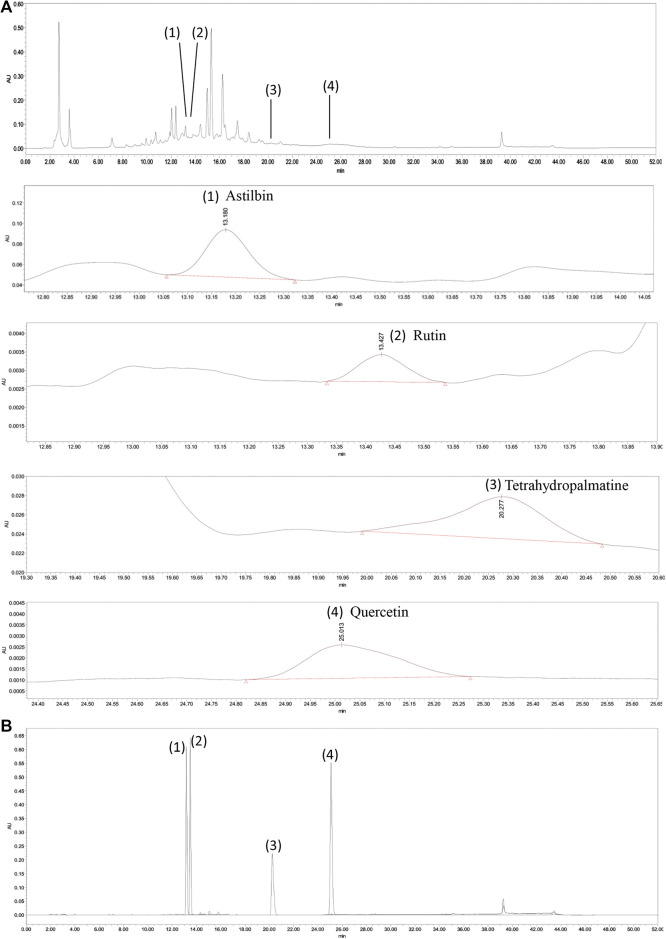
HPLC analysis of Qu-zhuo-tong-bi decoction (QZTBD). QZTBD **(A)** and standard substance **(B)**. Identification of main compounds in QZTBD as follows: Astilbin (1), Rutin (2), Tetrahydropalmatine (3), and Quercetin (4).

The characteristic chromatogram of the standard and QZTBD were shown in Figure 1. The retention times of astilbin, rutin, tetrahydropalmatine, and quercetin were 13.180, 13.427, 20.277, and 25.075 min, respectively. Through the calculation of the regression curve, the concentrations of these four components in QZTBD were 0.1174, 0.0063, 0.0140, and 0.0109 mg/g, respectively (Table 2).

**TABLE 2:** Herbal retention times and sample contents of components in QZTBD.

Constituents	Retention times (min)	Sample contents (mg/g)
Astilbin	13.180	0.1174
Rutin	13.427	0.0063
Tetrahydropalmatine	20.277	0.0140
Quercetin	25.013	0.0109

### QZTBD Alleviated Gouty Symptoms Effectively

**FIGURE 2:**
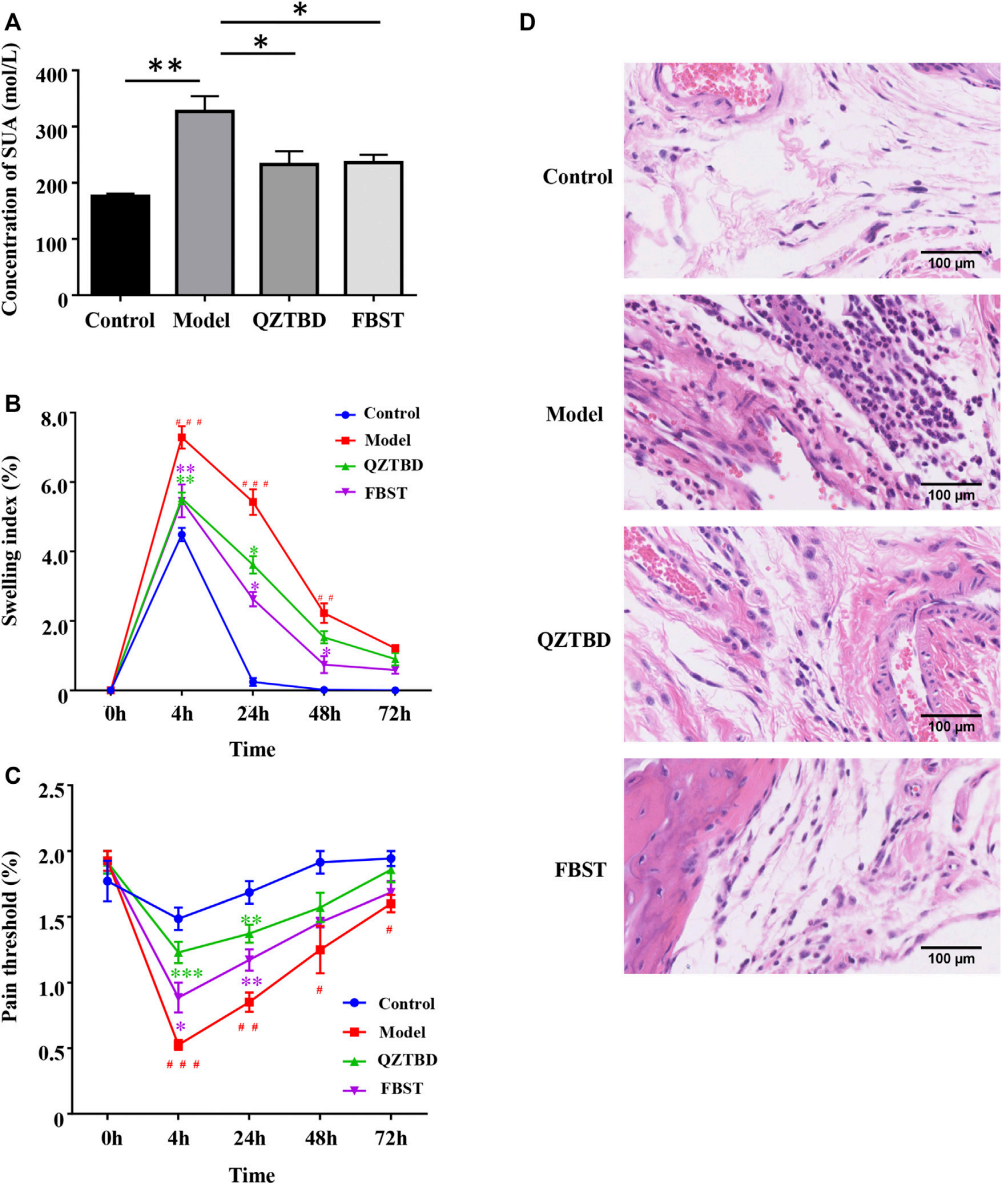
QZTBD alleviated gouty symptoms effectively. Effects of QZTBD on SUA **(A)**, footpad swelling index **(B)**, footpad pain threshold **(C)**, and HE analysis of the footpad **(D)**. “#” represents *p* < 0.05 in the comparison with the control group; “# #” represents *p* < 0.01 in the comparison with the control group and “# # #” represents *p* < 0.001 in the comparison with the control group; “*” represents *p* < 0.05 in the comparison with the model group; “**” represents *p* < 0.01 in the comparison with the model group and “***” represents *p* < 0.001 in the comparison with the model group. N = 7/group.

In order to evaluate the effects of QZTBD on gout mice, we monitored the animal’s SUA levels, footpad swelling degree, and pain threshold. Compared with the control group, the SUA level in the model group was significantly increased. As expected, both QZTBD and FBST (the drug commonly used for lowering SUA level and was used as a positive control) effectively decreased the level of SUA (Figure 2A). The animals that received an injection of MSU crystals showed an increase in footpad swelling and a decrease in mechanical pain threshold (Figures 2B,C). FBST showed a better performance in ameliorating MSU-stimulated swelling (Figure 2B), while QZTBD manifested a better anti-allodynic effect over the observation period (Figure 2C). HE analysis indicated that the model group demonstrated increased inflammatory cells’ infiltration and hyperplasia synovial. These pathological states were ameliorated to some degree by treatment with QZTBD and FBST (Figure 2D). There was no significant difference in spleen, kidney, liver indices, and CREA levels between the control group and the QZTBD group (Supplementary Figure S1), indicating the safety of QZTBD decoction on male C57BL/6 mice. These results suggest that QZTBD can effectively attenuate gouty symptoms induced by a high-fat diet and MSU injection without obvious toxicity.

### QZTBD Regulated the Microbial Community Structure

**FIGURE 3:**
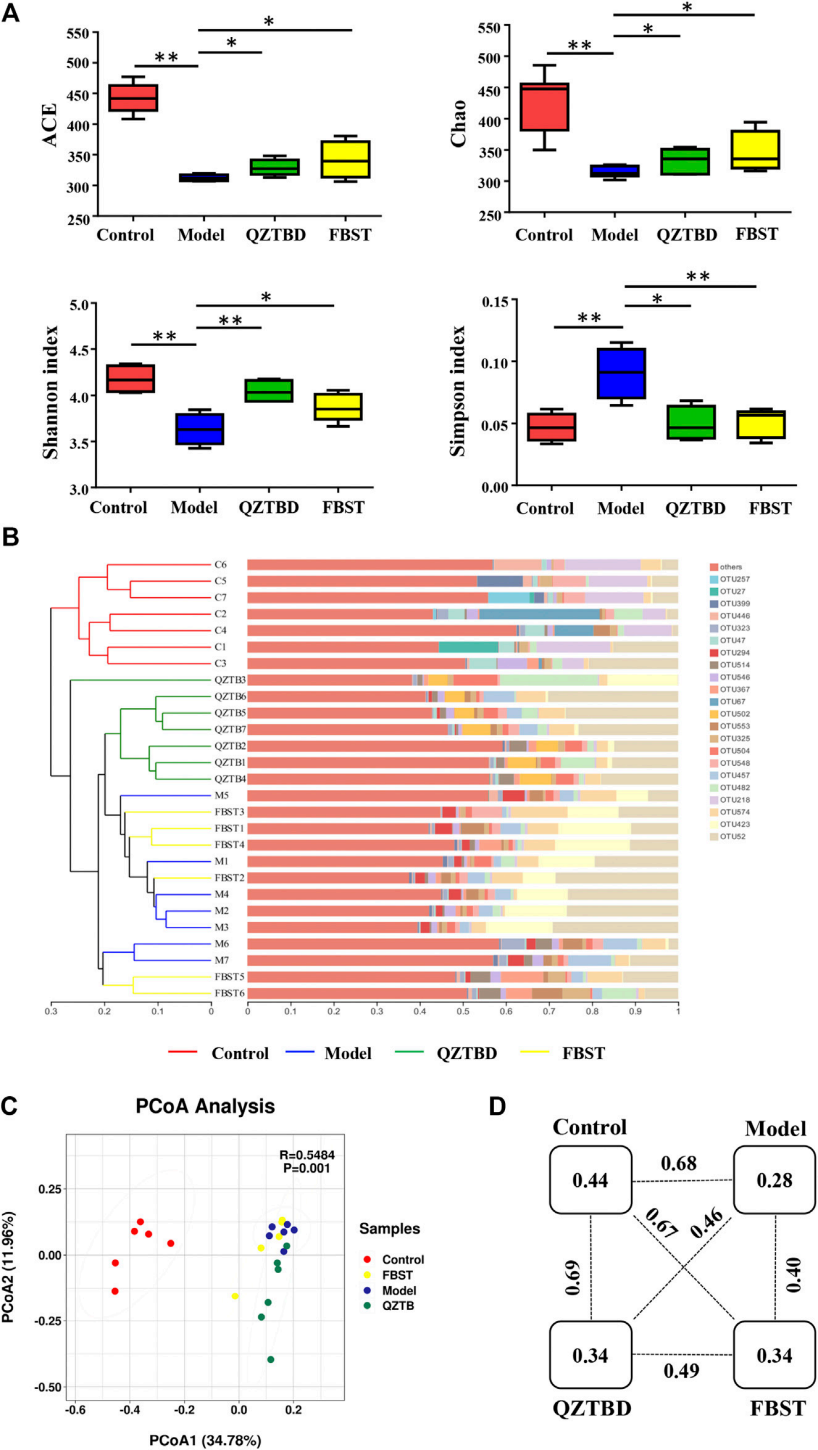
QZTBD regulated the microbial community structure. Effects of QZTBD on microbial richness and diversity **(A)**; Hierarchical clustering analysis on OTU level **(B)**; PCoA score plots **(C)** and distance between the two groups **(D)** based on Bray-Curtis dissimilarities. “*” represents *p* < 0.05; “**” represents *p* < 0.01. N = 7/group.

Mounting evidence has demonstrated that gut dysbiosis is a pivotal factor of gout and that TCM can regulate the composition and function of gut microbiota. We next sequenced the bacterial V3-V4 region of the 16S rRNA gene to profile gut microbiota composition between the normal and gout mice and evaluated the influence of QZTBD on the intestinal flora. A total of 1261461 tags were generated, with an average of 45,052 tags per sample. The rarefaction curves of all samples tended to be flat, indicating the amount of sequencing data is reasonable and the depth of sequencing is appropriate (Supplementary Figure S2). The estimated richness as indicated by ACE and Chao indexes were significantly lower in gout mice. Microbial diversity, which was assessed via Shannon and Simpson indexes, showed a robust decrease in the model group (Figure 3A). Both QZTBD and FBST could promote microbial richness and diversity. Hierarchical clustering analysis revealed FBST treatment mice have a similar microbial community to gout mice, while QZTBD-treated mice’s microbiomes are structured differently (Figure 3B). PCoA analysis (Figure 3C) showed that samples could be separated based upon the microbial community structure. Figure 3D demonstrated the distance between the two groups. The intragroup distance was smaller than the intergroup distance, indicating the microbiota composition of mice within the same group is more similar to mice from a different group. QZTBD can regulate the structure of intestinal flora in a unique way.

### Key Phenotypes Responding to the QZTBD Treatment in Gout

**FIGURE 4:**
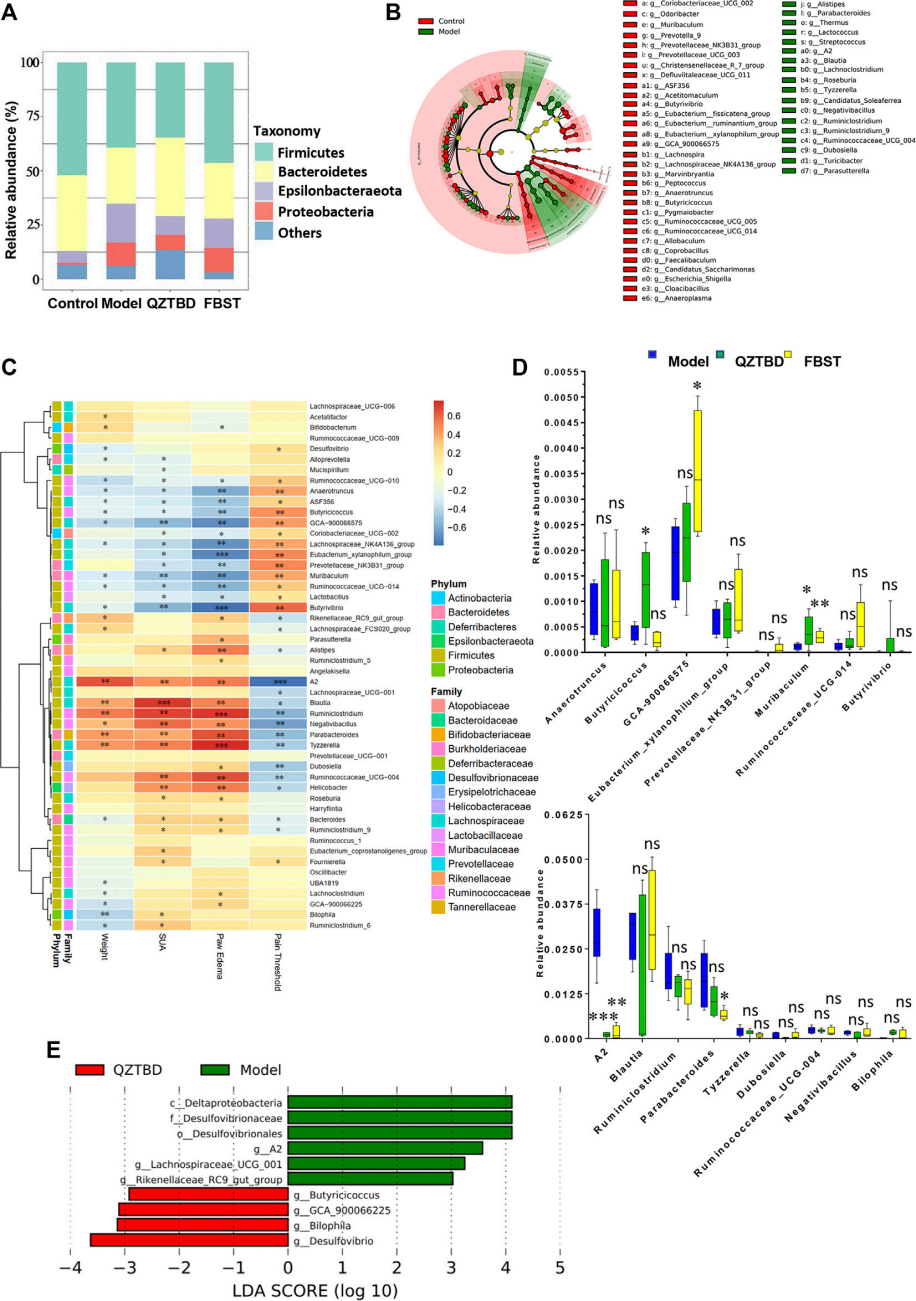
Key phenotypes responding to the QZTBD treatment in gout. Differences of gut microbiota at the phylum level **(A)**; LEfSe analysis between the control group and the model group **(B)**; Analysis of correlation between characteristic intestinal flora and gout symptoms is shown through a heatmap. Positive correlations are displayed in red and negative correlations in blue. The intensity of the color is proportional to the correlation coefficient **(C)**; Relative abundance of characteristic intestinal flora after QZTBD and FBST treatment **(D)**; LEfSe analysis between Model group and QZTBD group **(E)**. “*” represents 0.2 < *r* ≤ 0.4; “**” represents 0.4 < *r* ≤ 0.7; “***” represents *r* > 0.7 in Figure 4C. “ns” represents not significant; “*” represents *p* < 0.05, “**” represents *p* < 0.01 and “***” represents *p* < 0.001 in Figure 4D. N = 7/group.

Based on 16S rRNA gene sequencing, we observed that Firmicutes, Bacteroidetes, Epsilonbacteraeota, and Proteobacteria are the four dominant phyla (Figure 4A). Compared to the control group, the relative abundance of Firmicutes and Bacteroidetes decreased in the model group, while the amounts of Epsilonbacteraeota and Proteobacteria increased. QZTBD restored the abundance of Bacteroidetes, but FBST mainly altered the abundance of Firmicutes, suggesting these two drugs have different regulatory mechanisms (Figure 4A). LEfSe analysis showed that 31 bacterial genera were depleted in gout mice, while 18 genera were enriched in these same samples (Figure 4B). It is worthy to note that the abundance of SCFAs-producing bacteria was significantly reduced in the gut of gout mice (Figure 4B). A series of Spearman’s correlations were further conducted to elucidate the association between characteristic flora and symptoms of gout. As shown in Figure 4C, *Butyrivibrio*, *Butyricicoccus*, *Lachnospira*, *Eubacterium,* and *Faecalibaculum*, which are known as butyrate producers, were negatively correlated with SUA level and paw edema, while they were positively correlated with pain threshold. The bacteria those cultivated in gout mice were perfectly positively correlated with gout assessment indicators. The above results propose the importance of gut flora, particularly butyrate-producing bacteria, in the pathogenesis of gout. These taxa provide a new potential treatment target for gout. We next checked the changes of these characteristic flora after drug treatment (Figures 4D,E and Supplementary Figure S3). Both QZTBD and FBST inhibited the growth of *Lachnospiraceae*_A2 (a bacterium enriched in gout mice) and boosted the abundance of *Muribaculum* (a bacterium strongly correlated with SCFAs). Moreover, QZTBD notably increased the abundance of *Butyricicoccus* while FBST mainly produced an increase in abundance of *Lachnospiraceae*_GCA-900066575. These differences in genera compound our earlier finding that QZTBD and FBST play different roles in gut microbial regulation.

### QZTBD Influenced the Fecal SCFAs Levels

**FIGURE 5:**
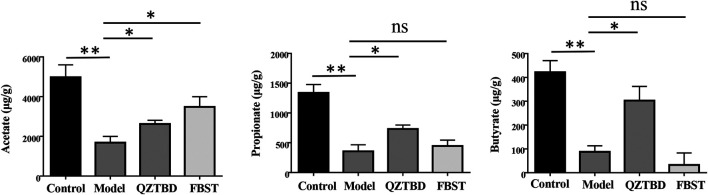
QZTBD influences the fecal SCFAs levels. Acetate **(A)**, Propionate **(B)** and Butyrate **(C)**. “ns” represents not significant; “*” represents *p* < 0.05 and “**” represents *p* < 0.01. N = 7/group.

Taking into account the decline of SCFAs/butyrate-producing bacteria, we next tested the concentration of the most abundant SCFAs, including acetate, propionate, and butyrate, in the feces of mice by GC-MS (Supplementary Figure S4). As shown in Figure 5 and Supplementary Table S2, as compared with the control group, the concentrations of acetate, propionate, and butyrate in the model group were dramatically decreased (*p* < 0.01). QZTBD significantly increased the levels of all these three main SCFAs, especially of butyrate (*p* < 0.05). FBST clearly improved fecal acetate level (*p* < 0.05), but its application had no effect on the contents of propionate and butyrate. These metabolic features were compatible with the results of microbial composition. They further demonstrated that abnormally reduced intestinal levels of SCFAs or SCFA-producing bacteria are usually closely related to gout, which has been commonly found in patients who were diagnosed with inflammatory bowel disease, type 2 diabetes, obesity, and autoimmune diseases.

### QZTBD Improved Intestinal Mucosal Barrier and Intestinal Urate Excretion

**FIGURE 6:**
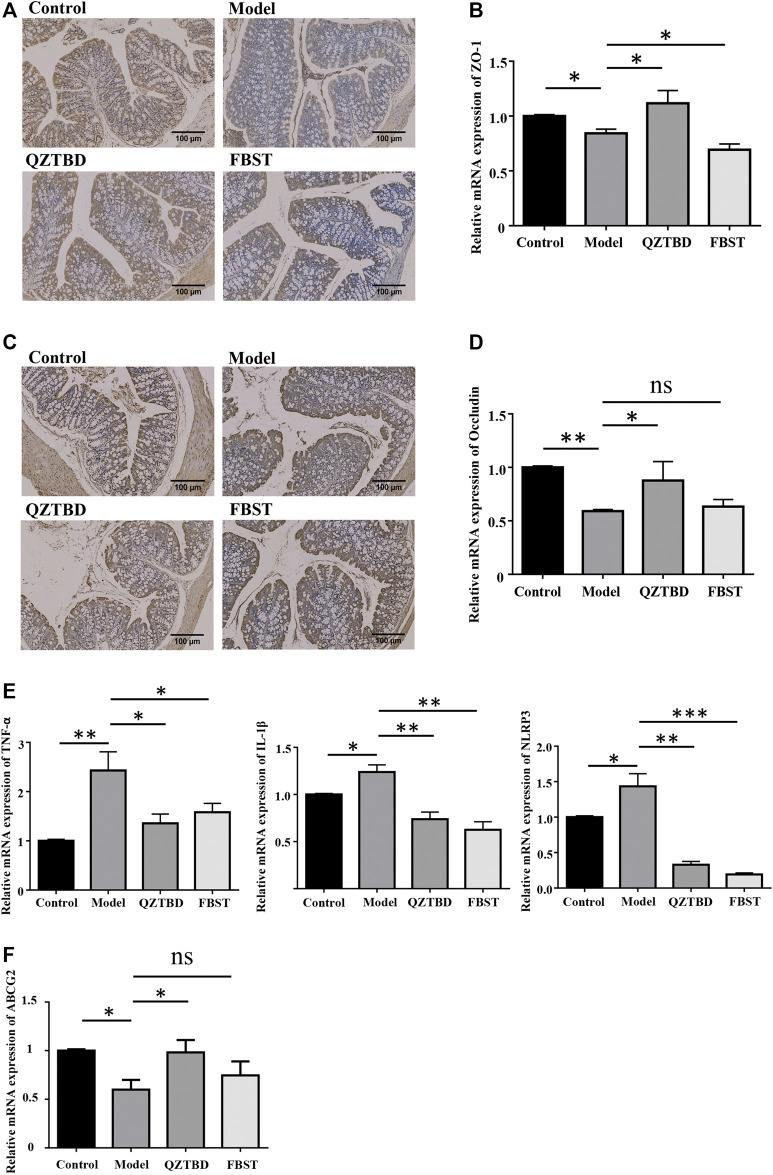
QZTBD improved intestinal mucosal barrier. Expression of ZO-1 in the colon was measured by immunohistochemistry **(A)** and *q*RT-PCR **(B)**; Expression of Occludin in the colon was measured by immunohistochemistry **(C)** and *q*RT-PCR **(D)**; The mRNA expression of inflammatory factors in the intestinal tissues **(E)**; The mRNA expression of ABCG2 in the intestine **(F)**. “ns” represents not significant; “*” represents *p* < 0.05, “**” represents *p* < 0.01 and “***” represents *p* < 0.001. N = 7/group.

Considering SCFAs are essential for the maintenance of the intestinal mucosal barrier function, the reduction of SCFAs may have an impact on the permeability of the intestinal mucosa by modulating the expression of tight junction (TJ)-related proteins. The levels of ZO-1 and Occludin were determined by IHC staining and *q*PCR. Consistent with our expectations, both protein and mRNA expression levels of ZO-1 (Figures 6A,B) and Occludin (Figures 6C,D) in the colon were significantly reduced in the model group. QZTBD up-regulated both mRNA and protein levels of these two TJ-related proteins. However, no significant expression change was detected in FBST-treated mice. Given the association between intestinal barrier dysfunction and inflammation, we next examined the expression of related inflammatory factors in intestinal tissues. The mRNA levels of NLRP3, IL-1β, and TNF-α in the model group were remarkably higher than those in the control group, and dramatically fell in QZTBD group. FBST also lowered the mRNA expression of NLRP3, IL-1β, and TNF-α despite its inability to repair the intestinal barrier (Figure 6E). These results suggest that QZTBD enhances intestinal barrier function by increasing the expression of TJ-related proteins and inhibiting intestinal inflammation. Furthermore, mRNA expression level of ABCG2, a well-known urate transporter, was decreased in the gouty mice. QZTBD increased the expression of ABCG2 and improved the intestinal urate excretion (Figure 6F).

### QZTBD Modulated the Expression of GPR43 and Energy Homeostasis in the Intestine

**FIGURE 7:**
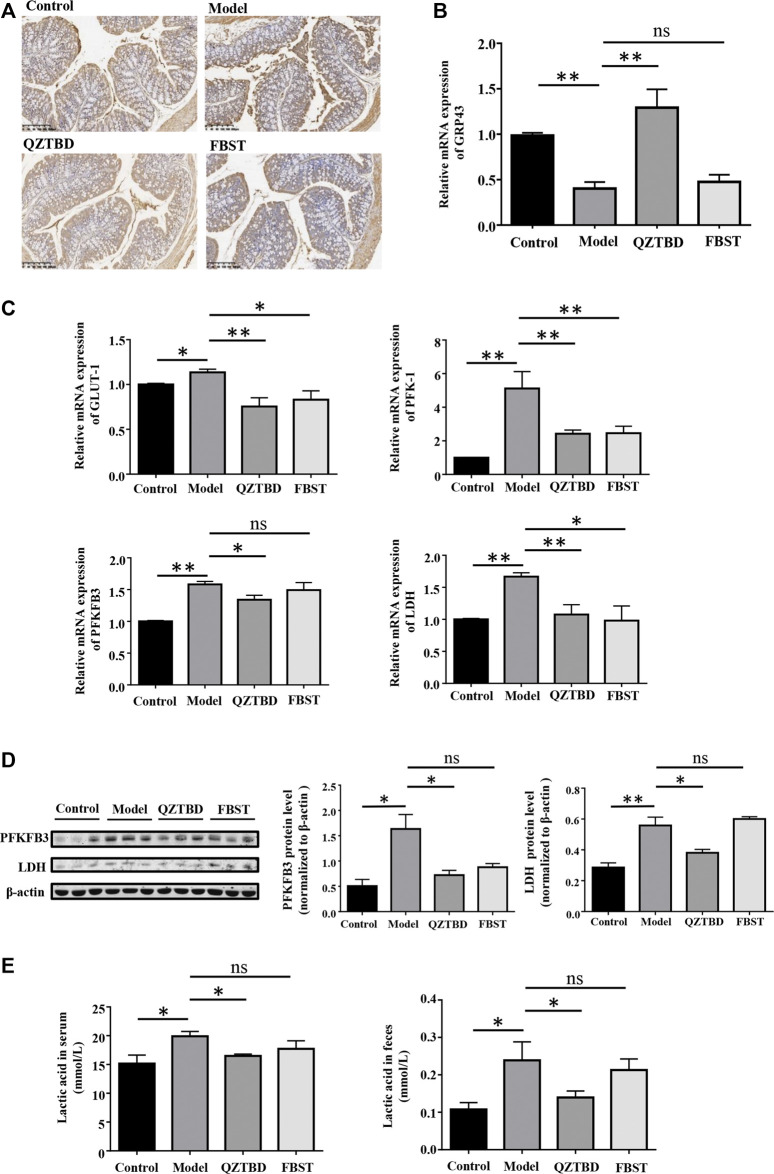
QZTBD modulated the expression of GPR43 and metabolic phenotype. Expression of GPR43 in the colon were measured by immunohistochemistry **(A)** and *q*RT-PCR **(B)**; The mRNA expression of key glycolytic enzymes (GLUT1, PFK1, PFKFB3, and LDH) in the intestinal tissues **(C)**; The protein expression of PFKFB3 and LDH in the colon were measured by Western blotting **(D)**. The concentration of lactic acid in serum and feces **(E)**. “ns” represents not significant; “*” represents *p* < 0.05, “**” represents *p* < 0.01 and “***” represents *p* < 0.001. N = 7/group.

Given the effect of SCFAs is derived from their ability to stimulate G-protein coupled receptors, we proceeded to examine the expression of GPR43. As described in Figures 7A,B, mRNA and protein levels of GPR43 in the colon were reduced in the gouty mice. QZTBD enhanced both mRNA and protein levels of GPR43 while FBST was unable to restore the levels of GPR43. On the other hand, mounting evidence has demonstrated that SCFAs are the primary energy sources for colonocytes and SCFAs alter the metabolic rate by stimulating GPR43. To reconcile these findings, we next examined the metabolic phenotype of intestinal cells. An elevated expression of glucose transporter type 1 (GLUT 1), which augments glucose uptake and glycolytic flux, was observed in gouty mice (Figure 7C). mRNA levels of key genes related to glycolysis, including PFK1 (phosphofructokinase 1), PFKFB3 (6-phosphofructo-2-kinase/fructose-2,6-bisphosphatase-3), and LDH (lactic acid dehydrogenase), were upregulated in the model group (Figure 7C). Western blotting analysis further verified the upregulation of PFKFB3 and LDH protein levels in gouty mice (Figure 7D). Moreover, the concentration of lactic acid, the dead-end product of glycolysis, was increased in both serum and feces, revealing the association of glycolysis and gout (Figure 7E). Both QZTBD and FBST inhibited the expression of GLUT1, PFK1, and PFKFB3, yet they resulted in different impacts on gene expression of LDH and lactic acid level (Figures 7C–E). We therefore speculated that SCFA deficiency is closely tied to enhanced glycolysis and increased susceptibility to gout. Inhibition of glycolysis is a potential target for gout treatment and QZTBD can effectively down-regulate the expression of glycolysis related enzymes.

## Discussion

**FIGURE 8:**
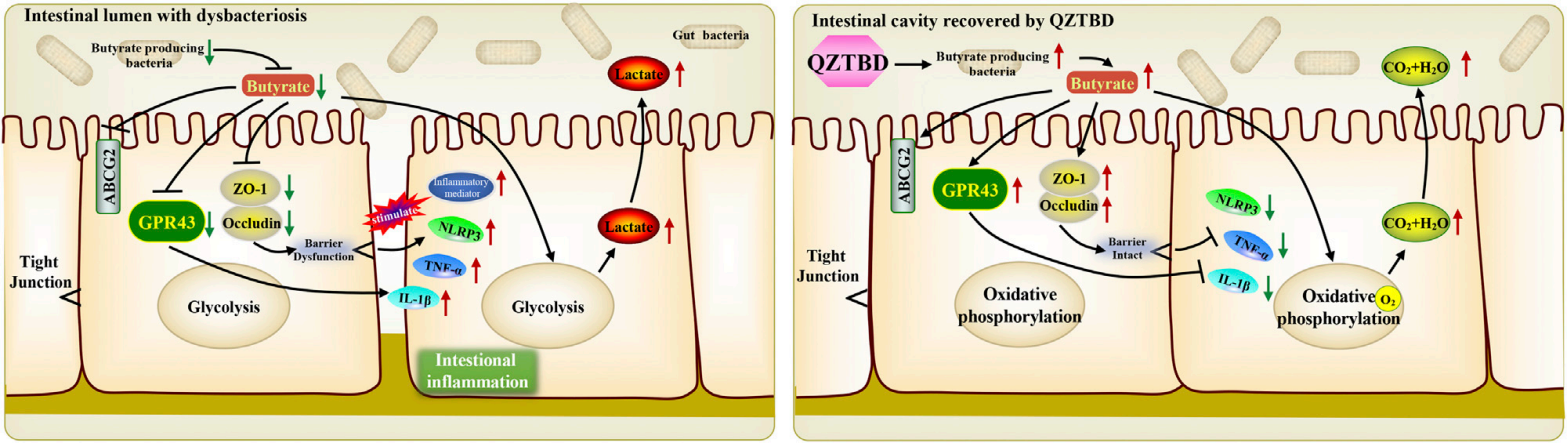
Proposed mechanism of QZTBD in the treatment of gouty arthritis. In gout mice, the deficiency of gut butyrate-producing bacteria resulted in the decrease of intestinal butyrate content. Dropped butyrate inhibited the expression of GPR43 and intestinal tight junction related proteins, and further shifted the metabolism of colonocytes toward glycolysis and activated downstream inflammatory pathways. QZTBD increased the level of microbiota-derived butyrate by promoting the proliferation of butyrate-producing flora, thereby further activating the expression of SCFAs receptor GPR43, increasing the expression of intestinal tight junction related proteins, facilitating the metabolism of colonocytes toward oxidative phosphorylation, inhibiting downstream inflammatory pathways and eventually ameliorating gout. Red arrow indicates upregulation, green arrow indicates downregulation.

QZTBD is an empirical prescription that has been developed for treating gouty arthritis for many years. Our previous research demonstrated that the potential anti-gouty arthritis effect of QZTBD could be attributed to the inhibition of the activation of the NLRP3 inflammasome and the production of downstream proinflammatory cytokines (Lv et al., 2019). The present study further verified its analgesic and anti-inflammatory effects and explored its potential mechanism from the perspective of the gut microbiome. We found that QZTBD can effectively reduce the symptoms of gout arthritis with comparable effects to FBST. It does so without obvious harmful side effects on animals, suggesting its clinical value for gout treatment. Our results indicated that QZTBD may exert its therapeutic effects by restoring the composition of gut microbiota and promoting the generation of SCFAs. Through these changes, QZTBD treatment attenuates intestinal mucosal barrier function, prompts intestinal uric acid excretion, inhibits glycolysis, and suppresses the production of intestinal pro-inflammatory cytokines (Figure 8).

Uric acid is the final metabolite of purine metabolism in the human body and the homeostasis of uric acid is achieved by regulating the production, degradation, and excretion of uric acid (Zhang et al., 2018). The intestine has been proven to be an important place for urate excretion (Ichida et al., 2012), and accumulating evidence suggests that the gut microbiome is involved in the pathogenesis of gout (Ma, 2020). Initiation of responses to MSU crystal in gout model required microbiota, and germ-free mice or mice treated with antibiotics had no response to the injection of MSU crystals (Vieira et al., 2015). Intestinal flora usually affect purine metabolism by secreting metabolic enzymes such as xanthine oxidase (Chiaro et al., 2017), or producing specific metabolites such as SCFAs (Haslberger and Terkeltaub, 2015). A remarkable reduction of *Faecalibacterium prausnitzii* and the inhibition of butyrate biosynthesis was observed in gout patients (Guo et al., 2016). Our previous study showed that the genera *Lachnospiraceae* NC2004 group, *Lachnospiraceae* UCG_005, *Ruminococcaceae* NK4A214 group, and *Ruminococcaceae* UCG_011, which are associated with SCFAs production, were depleted in gout patients (Shao et al., 2017). In this study, we verified the depletion of butyrate producers in gout mice, which were strongly inversely correlated with symptoms in gout mice. Targeting the gut microbiome, especially SCFAs producing bacteria, is a potential novel strategy for characterizing and treating gout (Ma, 2020). QZTBD can restore the gut microbiota ecosystem and promote the growth of SCFA-producing bacteria such as *Butyricicoccus*.

SCFAs are important mediators that gut microbiota use to affect the host’s physiology and immunity. GPR43 is one of the primary receptors of SCFAs. Signaling through GPR43 plays a significant role in the anti-inflammatory effects of SCFAs (Maslowski et al., 2009; Haslberger and Terkeltaub, 2015). Viera et al. demonstrated that germ free mice show attenuated MSU crystal induced inflammation which was mediated by acetate, and acetate acts via the macrophage receptor GPR43 to modulate inflammasome activation and IL-1β production (Vieira et al., 2015). They also concluded that acetate alleviates the inflammatory response to MSU crystals by inducing caspase-dependent apoptosis of neutrophils and enhancing the production of anti-inflammatory mediators (Vieira et al., 2017). The study was limited to examining the effects of butyrate on gout. Cleophas et al. reported that butyrate inhibited the pro-inflammatory responses induced by the combination of urate crystals and the long-chain fatty acid palmitate (C16.0) in PBMCs from healthy donors and gout patients, acting as an inhibitor of class I HDACs (Cleophas et al., 2016). In this study, we found the concentrations of all three primary SCFAs in feces and the expression of GPR43 in the intestine were significantly decreased in gout mice. The inhibition of SCFAs-GPR43 signaling leads to exacerbated or unresolving inflammation in models of gout arthritis, which was observed in colitis and asthma models as well (Maslowski et al., 2009). QZTBD increased the production of SCFAs, especially the production of butyrate, and the expression of GPR43. The regulation of SCFAs-GPR43 signaling was one of the critical ways for QZBTD to alleviate gouty arthritis, which was different from the means by which FBST takes effect.

Although increased intestinal permeability has been reported to trigger an immune response and low-grade inflammation, disrupted intestinal barrier function is associated with the development of various diseases, including intestine inflammatory diseases, extra-intestinal autoimmune diseases, and metabolic disorders (Chelakkot et al., 2018; Zaiss et al., 2019). We are the first to report on the altered expression of intestinal TJ-related proteins in mice with gouty arthritis. Gut microbiota and their metabolites are closely related to intestinal barrier function. Eeckhaut et al. reported that patients with inflammatory bowel disease have lower numbers of *Butyricicoccus* bacteria. Administration of this bacteria result in a protective effect by strengthening the intestinal barrier function (Eeckhaut et al., 2013) and lowering intestinal TNF-α and IL-12 levels. Butyrate could prompt improvements to intestinal barrier function by inducing the expression of tight junction proteins such as ZO-1 and Occludin (Zaiss et al., 2019). Our results demonstrated that QZTBD enhances the expression of TJ-related proteins and inhibits the production of inflammatory cytokines by promoting the growth of *Butyricicoccus*. Moreover, it was reported that butyrate could upregulate the expression of urate transporter ABCG2 (Ferrer-Picón et al., 2020). Deficiency of butyrate is associated with the dysfunction of ABCG2. QZTBD increased the ABCG2 expression in the intestine, suggesting ABCG2 is a potential therapeutic target of QZTBD.

Another interesting aspect regarding the enhancement of glycolysis contributes to the development of gout. Glycolysis is a metabolic pathway characterized by low oxygen consumption, high glucose utilization, and high lactate release (Litvak et al., 2018). A very recent study reported the observation that MSU crystals lead to a metabolic rewiring toward the aerobic glycolysis pathway because of an increased expression of GLUT1 in the plasma membrane and an up-regulation of glucose uptake on macrophages (Renaudin et al., 2020). In our experiment, besides the increased expression of the key enzymes related to glycolysis (GLUT1, PFK1, PFKFB3, and LDH), the concentration of lactic acid was also enhanced in both feces and serum, indicating an increase in glycolysis in the mice model with gouty arthritis. Furthermore, there was a significant reduction of oxygen consumption and an obvious increment of HIF-1α expression in the intestine of gout mice, which further confirmed that the alteration of gut microbiota and intestinal metabolic phenotype associated with the development of gout (Supplementary Figure S5A). The inhibition of the glycolysis pathway has been proposed as a new target for the treatment of gout flare and other IL-1β-related inflammation (Wen et al., 2012; Mian et al., 2019). QZTBD effectively inhibits the expression of genes involved in glycolysis, reduces the concentration of fecal and serum lactate, and restores the intestinal oxygen consumption (Supplementary Figure S5B), revealing a possible biochemical mechanism underlying this clinical prescription.

## Conclusion

Our current results demonstrated that the anti-gouty arthritis effect of QZTBD, an empirical TCM prescription in clinic and a potential therapeutic decoction for the prevention and treatment of gout, may be attributed to the restoration of gut dysbiosis and enhancement of SCFAs formation, the renovation of the intestinal barrier function, the suppression of the key glycolysis-related enzymes, and the inhibition of the production of inflammatory factors.

## Data Availability Statement

The datasets presented in this study can be found in online repositories. The names of the repository/repositories and accession number(s) can be found in the article/Supplementary Material.

## Ethics Statement

The animal study was reviewed and approved by Experimental procedures were approved by Laboratory Animal Management and Welfare Ethical Review Committee of Zhejiang Chinese Medical University (Permission number: ZSLL-2018-0012).

## Author Contributions

TS and XW were responsible for the design of the work. XW, YL, SS, ZH, JC, ZX, and XS were responsible for the acquisition and analysis of data. TS and XW drafted the manuscript. CW and TS approved the final version to be published.

## Funding

This research was supported by grants from the National Natural Science Foundation of China (81873145 and 82074248 and 81873269).

## Conflict of Interest

The authors declare that the research was conducted in the absence of any commercial or financial relationships that could be construed as a potential conflict of interest.
