# Supplementary material for: Qu-Zhuo-Tong-Bi Decoction Alleviates Gouty Arthritis by Regulating Butyrate-Producing Bacteria in Mice
Source: Front Pharmacol. 2021 Feb 2;11:610556. doi: 10.3389/fphar.2020.610556 (PMC7884811; doi:10.3389/fphar.2020.610556)
Supplement: Supplementary file 1 [file datasheet1.docx]

Qu-Zhuo-Tong-Bi Decoction Alleviates Gouty Arthritis by Regulating Butyrate-producing Bacteria in mice

Xianghui Wen ^1†^, Yu Lou ^1†^, Siyue Song ^1^, Zhixing He^1^, Juan Chen^1^, Zhijun Xie^1^, Xiaowei Shi^1^, Chengping Wen^1^^*^ and Tiejuan Shao^1*^

^1^College of Basic Medical Sciences, Zhejiang Chinese Medical University, 548 Binwen Road, Hangzhou, Zhejiang, 310000, China.

Supplementary Material

Table S1 *q*PCR amplification primer sequence

| Gene | | | Primer sequence (5' to 3') | Number of bases |
| --- | --- | --- | --- | --- |
| NLRP3 | F | | ATGTGGACACGGAAGAGACAA | 21 |
|  | R | | GGTAAGGGTAAGATTGGTGGTG | 22 |
| TNF-α | F | | GTGGTGCTCAGCTGCAAGTC | 20 |
|  | R | | TGTGGGTCTCAGGGAGATCAC | 21 |
| IL-1β | F | | TCATATCTTCAACCAAGAGGTA | 22 |
|  | R | | CAGTGAGGAATGTCCACAAACTG | 23 |
| GPR43    ZO-1  Occuldin  GLUT1  PFK1  PFKFB3  LDH  ABCG2 | | F  R  F  R  F  R  F  R  F  R  F  R  F  R  F  R | GGCTTCTACAGCAGCATCTA  AAGCACACCAGGAAATTAAG  GAGAGACAAGATGTCCGCCA  CCATTGCTGTGCTCTTAGCG ATGTCCGGCCGATGCTCTC  TTTGGCTGCTCTTGGGTCTGTAT  CTTGCTTGTAGAGTGACGATC  CAGTGATCCGAGCACTGCTC  GACGAATTCGCTTCCGCCCAGTCCAGC  GATCTCGAGCTAGCGACTCTTCCGGTG  GATCTGGGTGCCCGTCGATCACCG  CAGTTGAGGTAGCGAGTCAGCTTC  TATCTTAATGAAGGACTTGGCGGATGAG  GGAGTTCGCAGTTACACAGTAGTC  AAACTTGCTCGGGAACCCTC  CTCCAGCTCTATTTTGCATTCC | 20  20  20  20  19  23  21  20  27  27  24  24  28  24  20  22 |
| β-actin | F | | GGCTGTATTCCCCTCCATCG | 20 |
|  | R | | CCAGTTGGTAACAATGCCATGT | 22 |

Table S2 The changes of SCFA content (μg/g)

| Group | Acetate | Propionate | Butyrate |
| --- | --- | --- | --- |
| Control | 4978 ± 626.1 | 1337 ± 140.2 | 421.9 ± 48.89 |
| Model | 1680 ± 318.9 ^##^ | 356.1 ± 109.7 ^##^ | 87.78 ± 25.17 ^##^ |
| QZTBD | 2624 ± 182.5 * | 730.4 ± 66.73 * | 302.6 ± 59.86 * |
| FBST | 3483 ± 514.1 * | 445 ± 98.74 ns | 32.6 ± 50.38 ns |

“# #” represents *P* < 0.01 in the comparison with control group; “*” represents *P* < 0.05 in the comparison with model group; “ns” represents not significant. N = 7/group.


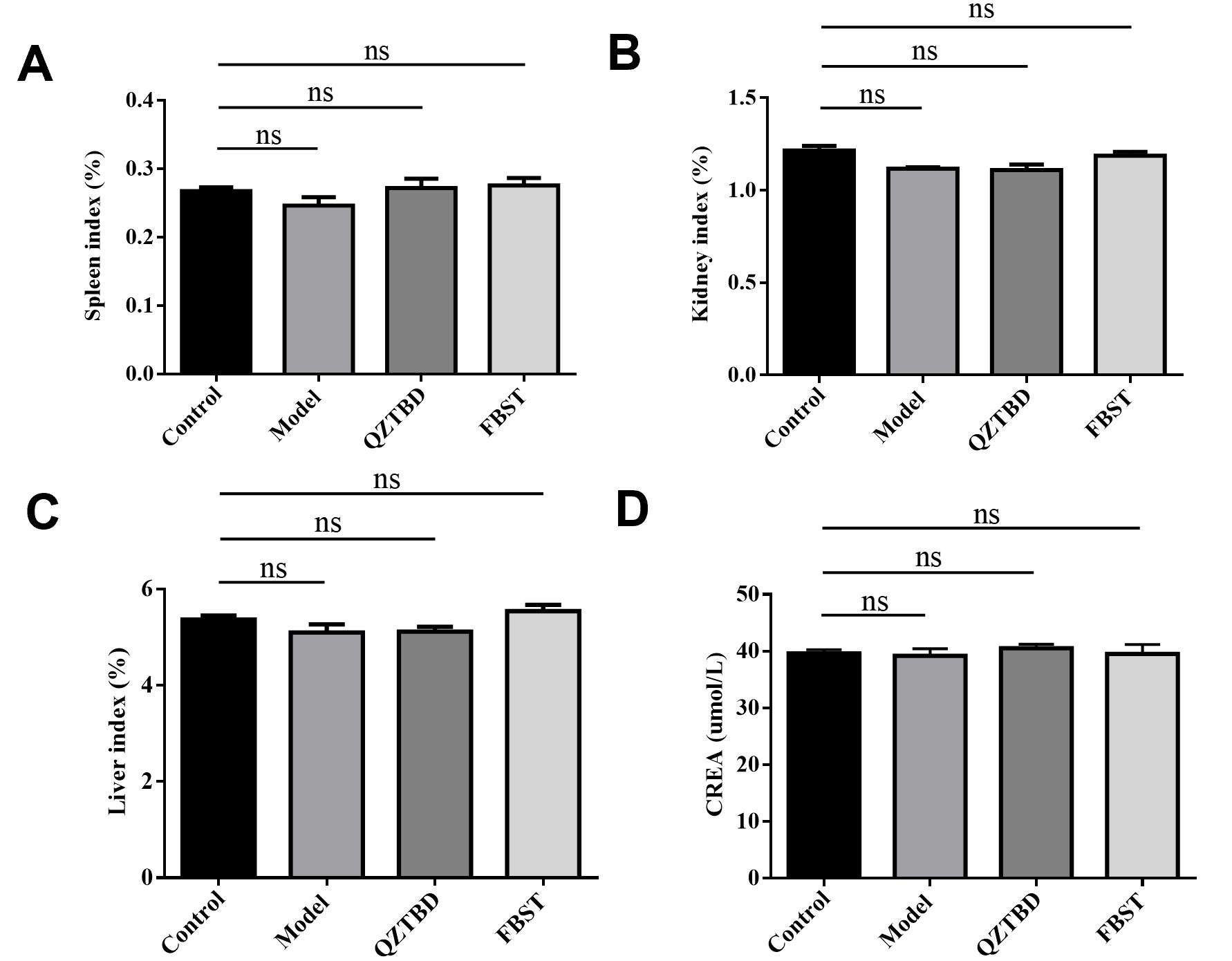


Figure S1 Effects of QZTBD on spleen index (A), kidney index (B), liver index (C), and CREA (D). “ns” represents not significant. N = 7/group.


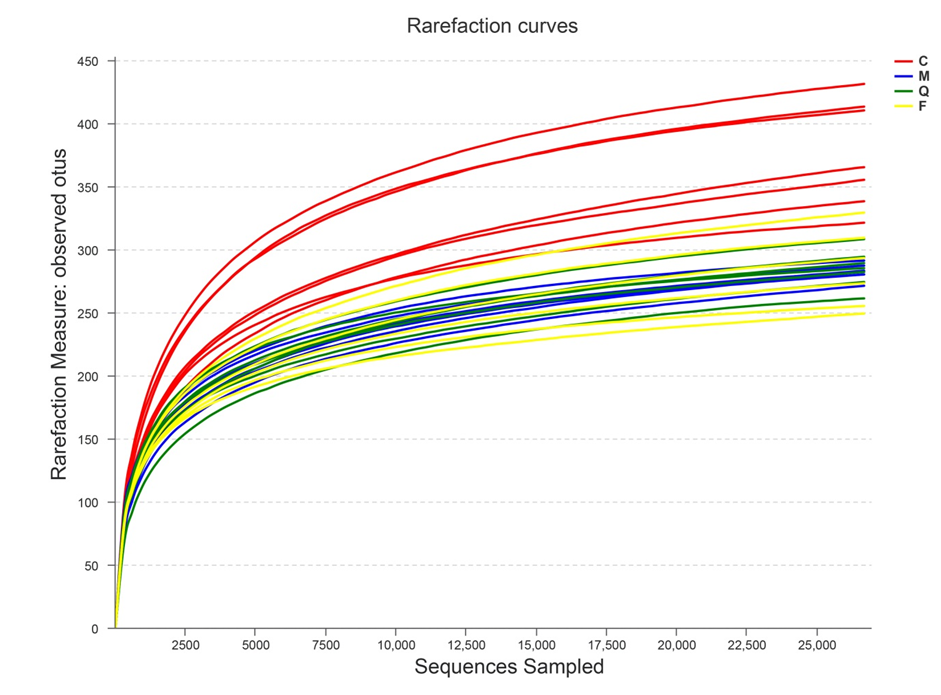


Figure S2 Rarefaction curves of the 16S rRNA gene reads based on OTUs at 97% sequence similarity. C(Control), M(Model), Q(QZTBD), F(FBST).


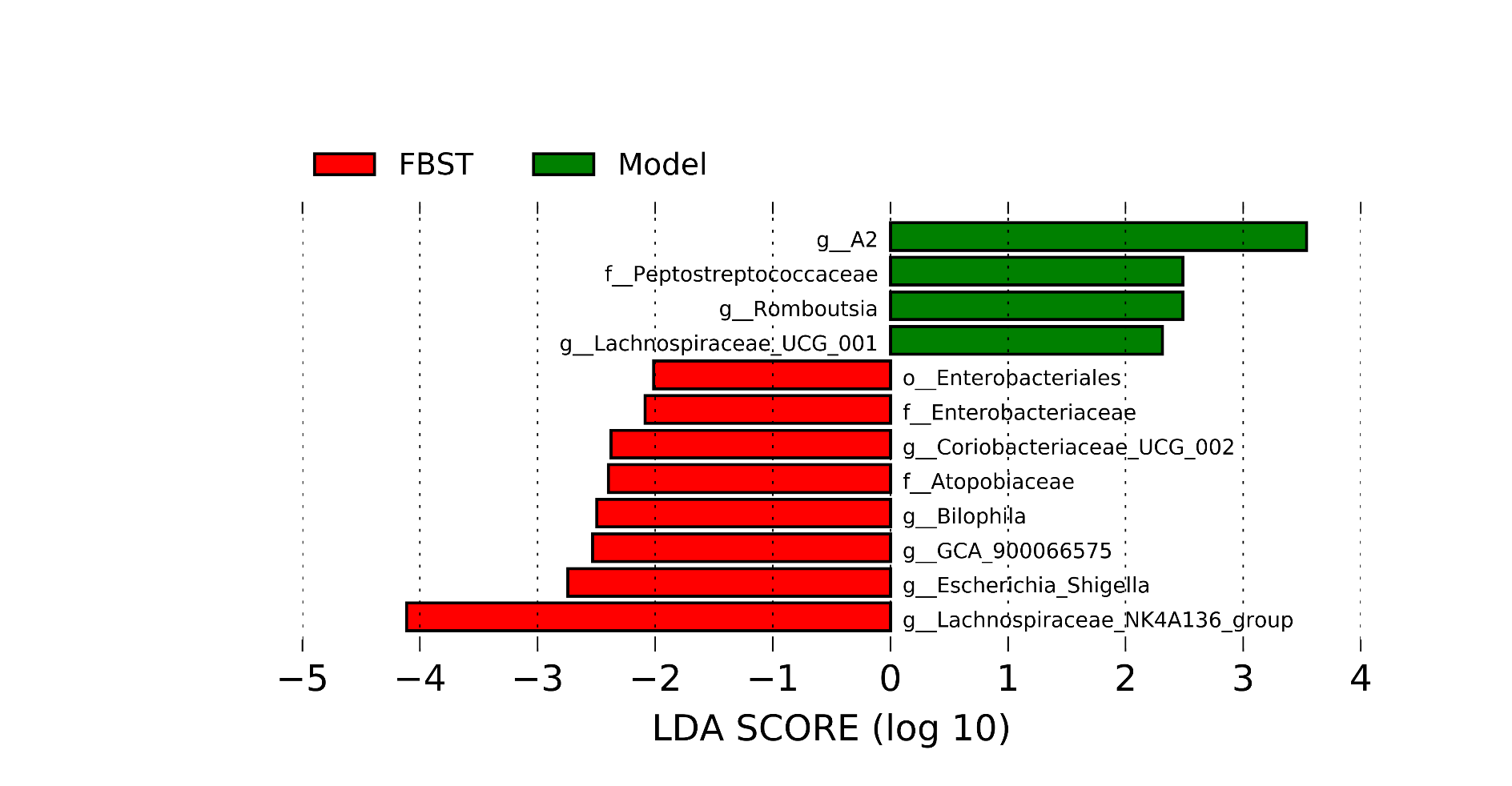


Figure S3 LEfSe analysis between Model group and FBST group.


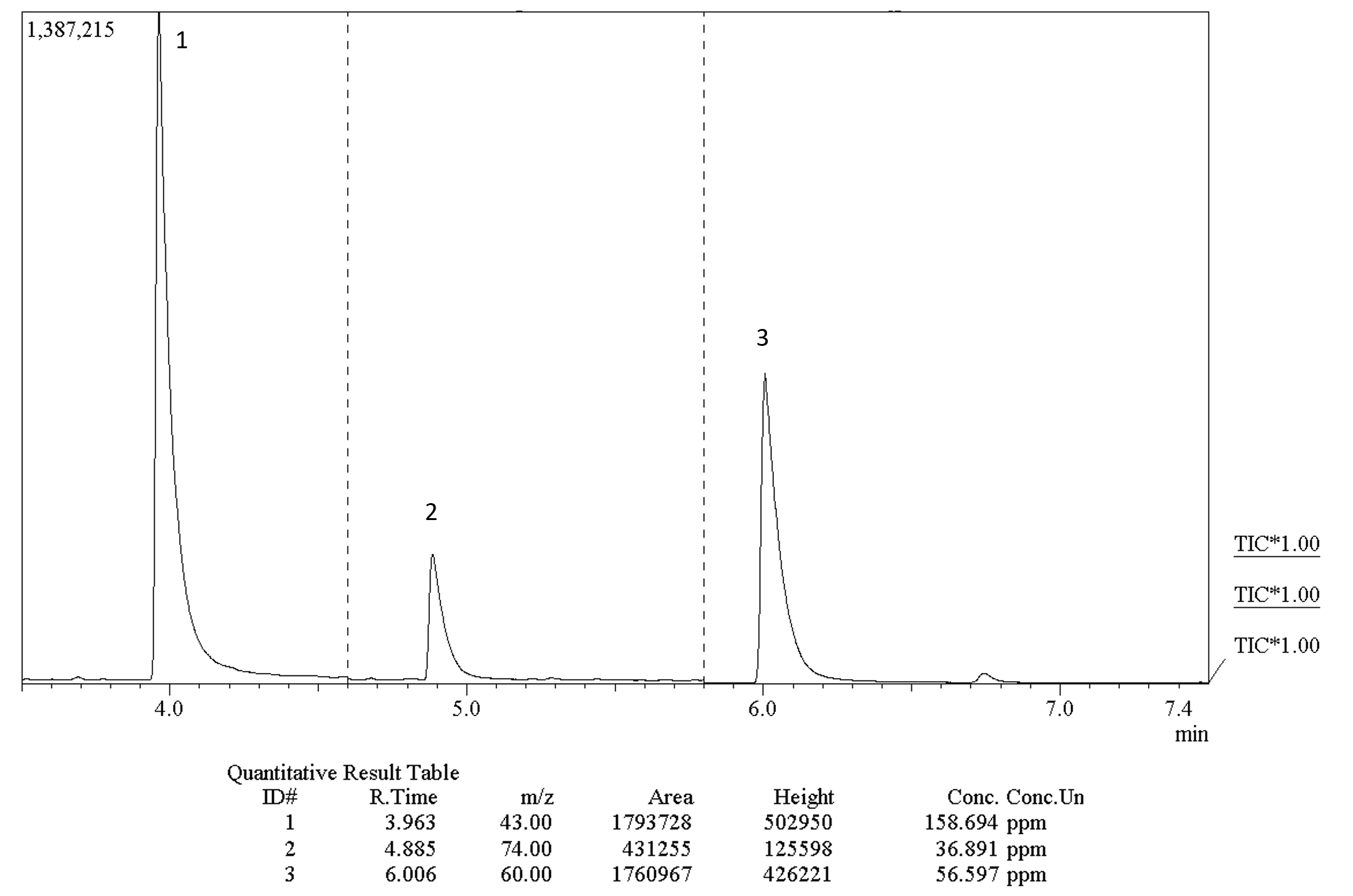


Figure S4 GC-MS characteristic chromatogram of faeces. Acetate (1), Propionate (2) and Butyrate (3).


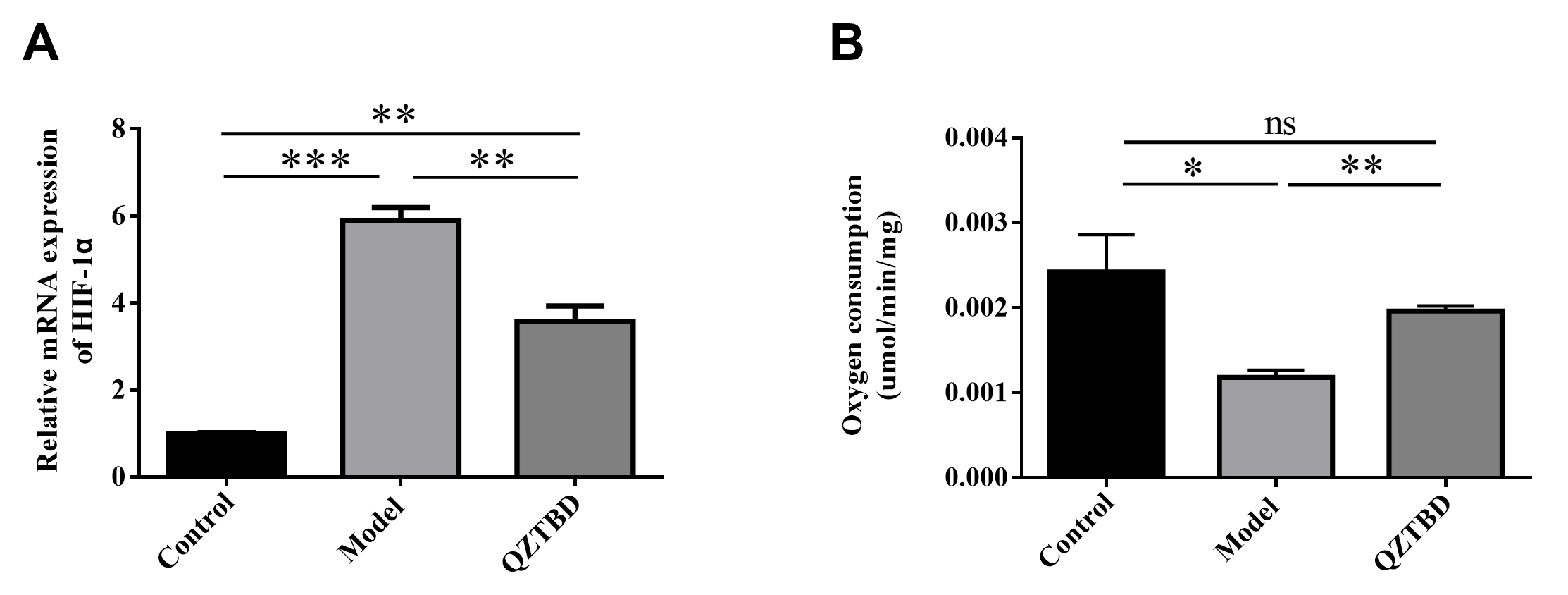


Figure S5 Relative mRNA expression of HIF-1α (A) and Oxygen consumption (B).

# Data availability statement

The raw data of 16S rRNA gene sequencing have been submitted to NCBI Project under accession number PRJNA656081 with NCBI Sequence Read Archive under accession number SRP276983.
